# Using Group Concept Mapping to Develop a Conceptual Model of Housing and Community-Based Residential Settings for Adults With Severe Mental Illness

**DOI:** 10.3389/fpsyt.2020.00430

**Published:** 2020-06-19

**Authors:** Amélie Felx, Mary Kane, Marc Corbière, Alain Lesage

**Affiliations:** ^1^Research Centre, Institut universitaire en santé mentale de Montréal (IUSMM), Montreal, QC, Canada; ^2^Mental Health and Addiction Programs, Douglas Mental Health University Institute, Montreal, QC, Canada; ^3^Concept Systems Incorporated, Ithaca, NY, United States; ^4^Department of Education and Pedagogy – Career Counselling, Université du Québec à Montréal, Research Centre of IUSMM, Montreal, QC, Canada; ^5^Mental Health and Work Foundation of IUSMM, Montreal, QC, Canada; ^6^Department of Psychiatry and Addictions, Faculty of Medicine, Montreal University, Montreal, QC, Canada

**Keywords:** housing, community-based residential settings, supported/supportive housing, concept mapping, mental health services, conceptualization, mixed-method approach

## Abstract

**Background:**

Most existing conceptual models of residential environments and housing programs were developed over a decade ago or lack comprehensiveness. The attributes to be used to describe housing programs with adequate specification remain unclear including the attributes that mediate service user outcomes. In this study, group concept mapping was used to develop a conceptual model of housing and community-based residential settings for adults with severe mental illness based on stakeholder perceptions and values.

**Methods:**

Participants were selected through purposive sampling and included service users, family members, staff working in residential facilities, mental health workers and managers. Participants (n=221) generated 1,382 statements describing attributes of housing and community-based residential settings for adults with severe mental illness (99% saturation). Thematic content analysis was conducted to analyze the statements and create a list of 140 selected statements. Participants then rated (n=416) and sorted into categories (n=73) the selected statements. Descriptive statistics were computed for each statement relative importance. Multidimensional scaling and hierarchical cluster analysis were used to produce the conceptual model (maps). Stakeholders (n=12) were also involved in the interpretation of the maps.

**Results:**

The findings show overall concordance between stakeholders in relation to statements perceived importance (rating) and the statements inter-relationships (sorting). The stress value of 0.23 indicates that the two-dimensional solution of the multidimensional scaling analysis fits the data set (goodness of fit). The final conceptualization includes 12 clusters: (1) A balanced and healthy housing system; (2) Quality and management practices (facility/local level); (3) Physical external environment; (4) Services tailored to needs and preferences; (5) Services and interventions provided (linkage); (6) Equality, policies and availability of activities; (7) Organizational structure and staff qualities; (8) Services and interventions (learning skills); (9) Services and interventions (daily living support); (10) Personal space and right to privacy; (11) Physical interior environment; (12) Respect, functioning and atmosphere.

**Conclusion:**

The results illustrate the multifaceted and multilevel nature of community-based residential settings through a visual representation. They articulate a number of attributes, clusters and dimensions that could be included in a common conceptual model of community-based residential settings and housing for adults with severe mental illness.

## Introduction

Community-based residential settings are widely implemented in Australia, Europe, and North America and constitute a costly key component of a modern mental health service system for adults with severe mental illness (1–3). Decades of research in the field have left clinicians, managers, and policy makers wondering what works best for specific group of service users, and why. Methodological weaknesses of relevant studies may partially account for the lack of guidelines and evidences, but the foremost and well-documented problem is variation: variation in terminology (1, 4, 5), variation in inputs and processes even within setting types (6, 7), variation in desired outcomes or functions, and variation in operationalization of housing models (8–10). Indeed, there is still ambiguity surrounding the description of these settings (1, 4, 11–13) using systematic attributes or features which are of importance to service user experiences and outcomes. Underlying the problem may be the fact that the field lacks an accepted framework for conceptualizing housing and community-based residential settings for adults with severe mental illness and for unifying decades of evolution in the field.

Housing and residential service models have evolved fueled by deinstitutionalization, by the move toward community integration and by recovery-oriented practice. Numerous studies since 1970 have highlighted potential key attributes of housing and community-based residential settings for adults with severe mental illness. Indeed, these settings have been described in terms of structural characteristics, process of care or both [e.g., Rog and Randolp multisite evaluation (6), *PROGRES* (14–16)*, UTOPIA-study* (17), *QuEST programme* (18)]. Studies comparing the outcomes of adults with severe mental illness living in different types of settings have mostly yield mixed results (1, 11–13, 19, 20) and have assessed various outcomes. Some noteworthy findings are that housing generally reduces days of homelessness and hospitalization and a trend for an inverse relationship between restrictiveness of setting and outcomes. A recent study conducted in England found a positive association between successfully moving on from mental health supported accommodation and two specific aspects of service quality: promotion of human rights and recovery-based practice. It also measured a lower quality of life in service users living in independent apartments with peripatetic support [see *QuEST programme* (21)]. Recent taxonomies can now be used to classify supported accommodation models, but they are, by definition, reductive and do not provide a detailed description of housing and community-based residential settings elements of care (4, 5, 22). Nor can they capture variation within service models. The tools used are also a great source of information on potential attributes and dimensions [e.g., *QuIRC-SA* (23), *COPES* (24)] but vary between studies. Unable to identify a comprehensive and systematic set of attributes to describe these settings from existing studies, we turned to models and frameworks to reach a better and in-depth understanding of community-based residential settings. In this vein, several models have been developed [e.g., (6, 7, 25–30)].

To our knowledge, only three models have provided a relatively comprehensive representation of the physical and socio-cultural attributes of housing environments and programs which could be critical to service user outcomes: (1) *The Ecological Framework for the Study of Community Housing for the Chronically Mentally Disabled* (26, 31); (2) *A Model of the relationship between program and personal factors and patient outcomes* (27); and (3) Kloos and Shah's (29) *Framework to study the ecology of housing environments* of persons with severe mental illness. All emphasize the complex and multifaceted nature of community-based residential settings. They go beyond the individual level to sharpen our understanding of this complex intervention as recommended by Tansella and Thornicroft (32). Each of the three models distinguishes two to four levels of attributes among: a person (level 1) with specific characteristics whom lives in a setting (level 2) located within a neighborhood and a community (level 3) and a region (level 4). Each model also uses domains that may influence service users to describe community-based residential settings and housing among: (1) the physical environment; (2) the social environment or system; (3) interpersonal relationships; (4) planning and policy (5) service delivery or support environment.

All of these three models are inspired by Moos's seminal work on treatment and human environments, which derived from several environments such as correctional facilities, families, and university residence halls (27, 33, 34). Moos's work as well as several researches in the 1960s, 1970s, and 1980s conceptualized inpatient and community-based residential settings for adults with mental illness based on social ecology and environmental psychology frameworks (25–27, 33–36). These studies provided us with a better understanding of the nature of service users' interrelations with their physical and socio-cultural environments (37), all components relevant to our understanding of residential environments. However, after revisiting those models and studies, two questions remained:

- Will the models and findings still apply decades later?
- Among the selected attributes, domains, and levels of the three models, which should be used to characterize the existing array of community-based residential settings for adults with severe mental illness?

Facing the challenge of evaluating community-based residential settings for adults with severe mental illness (e.g., schizophrenia) in Quebec, Canada, we recognized the need for deconstructing fully this complex component of the mental health service system into common attributes and dimensions. We consider the following principles to lead to a comprehensive conceptualization: (1) to use an inclusive approach therefore considering all potential attributes notwithstanding specific housing functions nor desired outcomes; (2) to use a bottom-up methodology therefore building on the knowledge of all relevant stakeholders; (3) to consider the large array of existing housing and community-based residential settings for adults with mental illness in Quebec.

This paper reports on the first phase of a research programme conducted in Quebec, Canada (38). Phase I was undertaken to articulate the multifaceted nature of housing and community-based residential settings as perceived by various stakeholders and then to develop a conceptual model. The results of Phase I shall be of interest to researchers for the validation/development of existing conceptual models and for the operationalization of housing and community-based residential settings, but also to the clinicians, patients, families and managers of residential facilities. Phase II aimed to develop a tool to describe housing ranging from 24-h staffed congregate setting to independent tenancy with peripatetic support and will be presented in a forthcoming publication.

## Materials and Methods

### Design

For the purpose of developing a conceptual model of housing and community-based residential settings we used a form of mapping approach: group concept mapping (GCM). GCM has evolved since its inception in the 1980s (39–41). It is a structured mixed-method participatory approach that incorporates group processes and multivariate statistical analyses (multidimensional scaling and hierarchical cluster analysis). It was well-suited because of the complex nature of community-based residential settings and the need to enable multiple stakeholders with different interests and expertise to articulate their thinking on the topic. GCM enables us to represent those ideas visually in a series of interrelated maps. GCM involves six major steps: (1) preparation; (2) generation of statements; (3) structuring of statements (rating and sorting); (4) data analysis and representation of statements; (5) interpretation of results; and (6) utilization of results and maps (39, 42). Steps 2, 3, and 5 involved participants.

### Setting

Generation of statements (step 2) involved four regions across the province of Quebec in Canada. Participants in the structuring of statements (step 3) came from five different regions. Sites were selected based on the available range of housing and community-based residential settings as well as catchment area (range in size and geographical spread) and location (urban, suburban, rural). The first four regions covered over 50% of public community-based residential facilities and 45% of the province population.

### Participants

All participants were selected through purposive sampling to maximize heterogeneity and to ensure that all major perspectives were represented (not in proportion to what exists in the population of participants). These included stakeholders with a variety of lived experiences representing the range of available housing models in Quebec. Participants were regrouped in four stakeholder groups: (1) service users; (2) family members; (3) managers, administrators of residential facilities or associations, and professionals supervising community-based residential settings; (4) staff working in various residential facilities or community associations and mental health workers. The four stakeholder groups were only mixed during the interpretation of the results (step 5). The inclusion criteria required participants to understand French and to be 18 years of age or older. Those who lacked capacity to provide an informed consent were not eligible. The research project was approved by seven ethics review boards, and all participants provided written informed consent.

The concept mapping process ultimately involved 722 incidents of participation as follows: 221 in generating statements (step 2); 416 in rating statements and 73 in sorting statements (step 3); and 12 in interpreting results (step 5). There were some overlaps between the participants involved in the different steps as they were systematically invited to take part in the following tasks of the GCM process. Most of the individuals who generated the statements through brainstorming also rated and sorted the statements. Therefore, the total number of unique participants is estimated to be 500.

**Table 1** shows the sociodemographic characteristics of the participants involved in the six steps of the concept mapping process. Sociodemographic characteristics were not systematically collected during the generation of statements (step 2). Complementary information indicates that the participants involved in step 2 were mostly female (136/221, 62%) and French-Canadian (210/221, 95%). They came from the public sector (172/221, 78%) as well as the private and non-profit/volunteer sectors (49/221, 22%).

**Table 1:** Participants in the group concept mapping process (number and sociodemographic characteristics).

Stakeholder groups and subgroups	No. of participants (per GCM steps)				Sociodemographic characteristics		
	Generation (Step 2)	Rating (Step 3a)	Sorting (Step 3b)	Validation (Step 5)	Age ^a^	Male (%)	Years (*sd*) ^b^
**Set 1 – Service users and family members**							
**U. Services users living in various settings (n (%))**	**49 (22.17)**	**172 (41.35)**	**7 (9.59)**	**1 (0.08)**	**46-50**	**50.5**	**15.68 (12.26)**
Independent living, supervised apartment		39	5	1			
Foster home		30	1	–			
Group residence, room and board, hostel		96	1	–			
Other (e.g., family)		7	–	–			
**F. Family members**	**43 (19.46)**	**11 (2.64)**	**1 (0.01)**	**1 (0.08)**	**61-65**	**9.1**	**20.72 (13.50)**
**Set 2 – Services providers and others**							
**M. Professionals & managers (n (%))**	**46 (20.81)**	**79 (18.99)**	**27 (36.99)**	**6 (50.00)**	**46-50**	**32.5**	**18.44 (11.34)**
Managers and administrators		34	12	2			
Professionals overlooking facilities		37	12	2			
Others (e.g., academic, agencies, NPO)		8	3	2			
**S. Staff including mental health workers (n (%))**	**83 (37.56)**	**96 (23.07)**	**25 (34.25)**	**3 (25.00)**	**46-50**	**29.2**	**12.93 (9.34)**
Foster home (operators)		24	8	1			
Group residence, supervised apartment, room and board, hostel (employees or unit managers)		72	10	2			
Others		–	7	–			
**Mental health workers (n (%))**		**58 (13.94)**	**13 (17.80)**	**1 (0.08)**	**41-45**	**31.58**	**15.80 (11.52)**
Case managers or mental health practitioners		54	11	1			
Peer support workers		4	2	–			
**TOTAL**	**221**	**416**	**73**	**12**			

a Median (ordinal scale).

b Years = number of years working in mental health field or using services.

U, Service users; F, Family members; M, Managers, administrators and professionals supervising residential settings; S, Staff working in residential facilities and mental health workers.

### Recruitment Process

The principal researcher (AF) first met with the management team coordinating access to public community-based residential settings in each region to present the research project and better understand the local housing estate and mental health services. They provided a list of all foster home caregivers and residential facility managers as well as a list of non-profit community-based residential facilities and housing, and whenever possible, private facilities. Potential participants received a letter explaining the purpose of the study and inviting their participation. At the commencement of the GCM process the principal researcher (AF) and a research assistant presented the project to managers, mental health workers, caregivers, and facility managers at each site. Follow-up meetings took place at each site to share information on the project progress. Selected participants were either volunteers, designed by the management team, identified by other participants or by the research team. Most service users were first identified by their case manager or mental health worker. Family members were mostly recruited *via* associations. We contacted each potential participant by telephone.

### Group Concept Mapping: The Six Steps in More Details

#### Step 1 Preparation

During the preparation step, the project was planned, and its logistics were determined. Participants were engaged and selected by the research team. The research team was multidisciplinary and included one person living in a residential facility and another with experience as a foster home operator. The research team decided not to use a web-based implementation because of the limited Internet access of many potential participants and their various literacy levels. Therefore, statements were generated (step 2) and rated (step 3) and results interpreted (step 5) during face-to-face group sessions. The sorting activity (step 3) was done manually by each participant *via* mail.

#### Step 2 Generation of Statements

Statements were generated during 13 live brainstorming sessions. Group sessions included from 4 to 28 participants. At the commencement of each session the principal researcher (AF) gave a description of the study and explained the brainstorming task. The general rules of brainstorming applied and no criticism of statements was allowed (43). Through one single prompt question, participants were asked to describe the attributes that community-based residential facilities and housing for adults with mental illness have or should have. Participants were given approximately 5 min to ponder. The facilitator then asked each participant for an idea/statement (or to pass his turn). After two or three rounds, hitch-hiking was used. Statements were fed directly into a computer and projected on a screen during sessions by the principal researcher (AF). Participants could visualize and validate the statements as they evolved. All statements were entered into a software package (ATLASti∕4.1).

Because of the large number of statements generated, thematic content analysis was used to reduce and analyze statements. A coding frame was developed by one member of the research team (AF) with supervision from two other researchers (AL and MC). The analysis was first conducted concurrently with group sessions to ensure that saturation was reached (99%). Then, an in-depth analysis was conducted. New codes and themes were added in the course of the analysis. Very good inter-rater agreement (91%) was achieved by two independent raters who coded 250 randomly selected transcripts. Intra-rater agreement was 99% after two months. Statements coded as residue were reviewed at the end of the analysis. The final list of statements to be used in the following steps of the GCM process comprised 140 selected unique statements (consisting mainly of quotes representative of relevant codes). Only codes related to structural and process elements were considered relevant in relation to our research questions. Before editing, statements were reviewed for singularity (reference to only one topic) and neutrality. Statements were left unchanged, whenever possible, to preserve as much as the content and wording in the original voice of the participants. The research team decided to keep a higher number of statements than usually found in GCM project to represent as accurately as possible the conceptual universe [generally a set of 80 to 100 statements can be reasonably processed in subsequent steps (39, 44)].

#### Step 3 Structuring of Statements

Information on the value and the relationships among the final set of statements was obtained. Because of the time required to conduct the tasks, rating and sorting were carried out separately. First, the rating of statements occurred during 41 group sessions (average duration of 2 h). At the commencement of each session, the principal researcher (AF) gave a short description of the study purpose and of its progress. Then, participants rated each of the 140 statement on two five-level ordinal scales: relative importance (1 = not important at all; 5 = very important) and current presence in the array of available housing (1 = not present; 5 = always present). Each statement was read by the facilitator and simultaneously projected on a screen. Statement numbers were listed in questionnaire form for participants to write down their answers. This procedure was chosen to ensure uniformity and because of discrepancies in participant literacy levels. The second task consisted of an unstructured card-sorting procedure (45). Each participant received one envelope containing instructions, 125 of the 140 statements printed on cards 3x8 inches, 20 blank cards and elastics. The 125 statements were randomly selected in an endeavor to reduce the burden associated with the sorting task. Participants were instructed to individually sort the statements into mutually exclusive piles in any way that made sense to them. They were instructed not to regroup all items in a single pile, not to have 125 piles (one pile per statement) and not to have a pile of miscellaneous statements. Participants also labeled each of the piles they created. Some participants reported taking over 5 h to complete the sorting task, which was described as very challenging despite the availability of unlimited telephone support and the effort made by the research team to reduce the number of statements (above the recommended number for GCM).

#### Step 4 Data Analysis and Representation of Statements

The investigators next computed the results from step 3 and selected those to be presented to participants for interpretation and discussion (step 5). Sorting and rating data were entered in The Concept System^©^ software (http://www.conceptsystems.com/content/view/the-concept-system.html) (46).

Ratings were also entered in a common database in the PASW Statistics 18 software (47). Descriptive statistics were computed. In order to compare stakeholders, two sets of stakeholders were created: S1 = service users (U) and family members (F) and S2 = staff and mental health workers (S) and professionals, managers and administrators (M). Mean difference for each statement perceived importance was computed using nonparametric tests (U de Mann-Withney) with Bonferronni correction (p = 0.0000). Results exclude participants with over 5% of missing data (> 5%). The rare random missing values were replaced by the attribute mean rating for the stakeholder subgroup (e.g., foster home caregivers).

In GCM, sorting results serve as an input to multidimensional scaling (MDS) and to the creation of maps. Each sort was transformed into an individual binary co-occurrence matrix, the number of which equals the number of completed sorts (n = 73) (Xij). For a single participant and for any two statements, the number placed in a matrix was 0 if the two statements (i and j) were not sorted together and was 1 if they were. Individual matrices were summed up across all participants to create a total 125 × 125 symmetric similarity matrix that indicated the number of participants that sorted two statements together (Tij) (39). This serves as a measure of the perceived conceptual closeness or distance between statements. Because the presence of both generic and smaller groupings (sorts) can create problems in the representation (48), one sort composed of only two clusters was excluded from the final analyses. Therefore, the analyses included the sorts of 72 participants (out of 73).

The total similarity matrix Tij was used as input for nonmetric MDS. The output is a geometric configuration of points (point map). The point map displayed the location of all brainstormed statements. The number of dimensions was limited to two (X1, X2) because two-dimensional configuration are generally easier to comprehend and in accordance with GCM guidelines (39, 44, 49, 50). The statements closer to each other on the map are expected to be more similar in meaning. Their proximity (distance) results from the fact that MDS placed them near each other because many participants sorted them together in piles. To gain a better understanding of the location of statements and determine the relative cohesiveness of the various parts of the map, especially the central area, bridging index were computed for each statement (0 to 1 scale) (39). An index closer to 1 indicates the statement more dispersed relationship to statements elsewhere on the map. As every statement must be placed on the map, the algorithm locates it in an intermediate position. An index closer to 0 indicates that a statement was placed by many participants with statements immediately adjacent to it on the map. Finally, Kruskal' stress value was computed to measure the degree to which the distances on the map are discrepant from the value in the input total similarity matrix (Tij). A high stress value implies that there is a greater discrepancy between the input matrix data and the representation of those data on the two-dimensional configuration. GCM projects are expected to have a stress value between 0.205 and 0.365 (M = 0.285) (39).

For each statement, the coordinate values (Xi1, Xi2) produced by the MDS analysis served as the input for an ascendant hierarchical cluster analysis using Ward's algorithm (51). Analysis partitioned the statements (dots) into a number of non-overlapping clusters (cluster map). The research team explored the suitability of several solutions (range of 5 to 18 clusters) based on practicality and interpretability. In GCM, there is no automatically mathematical criterion by which to select the final number of clusters. Starting with the 18-cluster solution we focused on the two clusters being merged to a point where the research team identified a lost in information. Cluster maps were also created for the four stakeholder groups to explore for agreements and disconnects.

#### Step 5 Interpretation of the Results

At this step, the representation of statements was presented to participants in accordance with the participatory nature of the process. All participants had been involved in one or more steps of the CGM process. The facilitator (AF) started the session with a brief reminder of the brainstorming, rating and sorting tasks performed previously. The computer-generated cluster map for all stakeholders (an aggregate of all the participants' individual representations) was presented cluster-by-cluster along with the 11 and the 13-cluster solutions. During the group session, the 12 participants were asked to comment on the number and on the content of clusters. They were also asked to place 15 additional statements on the map (125 sorted statements + 15 = 140 statements in all) to test the emergent commonly defined conceptualization. Clusters were labeled with their inputs during the group discussion.

#### Step 6 Utilization of Results and Maps

Concept mapping results (statements, attributes, clusters) were used to refine the conceptual model built on stakeholder perceptions and values. Indeed, the cluster and point maps were analyzed further in terms of dots and cluster location to create dimensions (axes). Principal components analyses (PCA) were conducted using current presence ratings to refine the model fit to the wide range of existing housing options in Quebec. A first draft of a tool describing the structural and process components of community-based residential settings for adults with mental illness was also developed based on the results. This tool was field-tested in various residential settings (38). The development of the instrument was part of the second phase (Phase II) of a research program and will be presented in a forthcoming manuscript.

## Results

### Attributes of Community-Based Residential Settings and Housing (Step 2)

The brainstorming sessions generated 1,382 statements (average = 106 per session; range = 86–148) describing the attributes of residential facilities and housing for adults with mental illness. These were grouped under the 236 codes, 50 sub-themes/codes and 9 themes of **Table 2**. The Table also indicates the number of sessions that a statement related to a sub-theme/code was mentioned and the stakeholder group to mention it. A larger dot indicates a higher occurrence of statements for a stakeholder group. No dot indicates no statement emerged.

**Table 2:** Attributes of community-based residential settings and housing for adults with severe mental illness generated by stakeholders (n=221) (step 2).

Themes	Sub-themes/codes	Codes or codes' descriptors	No.	Stakeholders			
				U	F	S	M
**Quality of care and management**	Philosophy of care and approaches	Share a common vision (recovery); inter-ministerial vision/policies; strengths model; push for supported housing; normalization User-centred approach (individualized)	8 11	• •	•	• •	● •
	Conflicting values/incoherence	Contradiction between approaches geared to recovery/citizenship and existing rules/regulations serving risk management.	9		•	•	●
	Evaluation and monitoring of residential facilities	Users' and families' satisfaction with services; service user and family participation; ongoing monitoring of quality; discrepancy between facilities; quality of unlicensed residential resources (lack of)	11	•	•	•	●
	Staff competencies	Qualifications; expertise/skills	11	•	•	•	•
	Ongoing staff training	Offer training to CRF staff (e.g., mental health)	8	•	•	●	●
	Diet	Serve balanced, quality food	11	●	•	●	•
	Burden on staff/families	Workload for families and staff/operators; ensure operators have days off (e.g., foster homes)	8	•	●	●	•
**Facing stigma**	Promotion	Communicate with and support landlords; public education; awareness	5		•	•	●
	Information about different CRF	Information for families, service users, staff and public; create websites with information on CRF	6	•	•	•	•
	Experience of stigma among service users, families, CRF and staff	Self-stigma; prejudice and discrimination from staff, communities and other service users against mental illness NIMBY; prejudice against caregivers and families	8 4	•	•	• •	• ●
**Local partnerships**	CRF staff	Involvement in setting goals and designing treatment plan; working jointly with mental health teams; lack of recognition	6		•	●	•
	Family members	Involvement in setting goals and designing treatment plan; access to information; lack of recognition	10	●	●	•	•
	Service users	Involvement in setting goals and designing treatment plan	5		•	•	•
	Community	Collaboration with community and local organizations (e.g., police force, supportiveness), access to resources	10	•	•	•	●
	Health services/network	Collaboration among stakeholders (CRF, family, service user, mental health team, community); continuity of care; access to emergency services	11		•	●	●
**Access to a variety** **of housing**	Admission process	No waiting list; simple process; well-defined and non-restrictive admission criteria; importance of meeting service user	11	•	•	●	●
	Evaluation	Evaluate service user's needs and abilities; consider service user's preferences/choices; ensure person-environment fit	13	•	•	•	•
	Types/range	Array; housing for specific sub-groups (e.g., youth, seniors, mothers, multicultural); crisis; respite; dynamic housing models (adapt to changing values and priorities) Specialized CRF for complex needs (e.g., forensic, substance abuse)	13	•	•	•	•
			11	•	•	•	●
	Access to propriety and independent living	Offer affordable/adequate housing (lack of); support access to tenancy; offer rent supplements	10	•	•	•	●
	Length of stay	Length of stay; *move on* versus *home for life*	11	●	•	•	•
**Outcomes**	Individual outcomes	Somewhere to live; feeling “at home”; being “at home”; security; community integration; isolation (independent living) A good life/quality of life; wellness	13 9	• ●	• •	• •	• •
**Support to stakeholders**	Operators/CRF staff	Informational (e.g., feedback, information, mentoring); tangible (e.g., regular supervision/visits, financial resources, night and weekend support, objectives/plan, training, volunteers); emotional (e.g., peer support); esteem (e.g., encouragement)	11	•	•	●	•
	Family members	Offer respite resources; offer sufficient services (lack of); peer/group support; training and education	10	•	●	•	•
**Physical environment**	Neighbourhood characteristics	Variety of locations (e.g., rural, urban); safety (e.g., crime, prostitution); density of CRF (normalization)	12	•	•	•	•
	Proximity to community resources	Usual services (grocery store, coffee shop, bank) and other services (e.g., hospital, community centre); transportation; parks	11	●	•	•	•
	Quality	Quality (e.g., plants, decoration); tidiness; home adaptations	12	•	•	•	•
	Equipment/material	Access to appliances (e.g., TV, computer, refrigerator); telephone	9	●	•	•	•
	Design	Smoking room; common areas; backyard; spaciousness; room(s) for staff; room for visitors	11	●	•	•	•
	Privacy	Private bathroom; private room; choice of sharing a room; keys (bedroom and home); unit access; quiet	12	●	•	•	•
**Social environment**	Residents' personal factors	Number of residents; heterogeneity; characteristics (e.g., skills, age, gender, motivation, dreams, functioning, symptoms, income)	13	•	•	•	•
	Staff	Staffing level; cover; turnover; categories (e.g., user-providers, professionals, paraprofessionals, back-up); qualities (e.g., believe in the person, flexibility, good listener, kindness, observe)	13	●	•	•	•
	Pets	Benefits of pets; pet permitted (or not)	6	•	•	•	•
	Atmosphere	Family-like; group living; respect; pleasant atmosphere (e.g., welcoming, warm, joyful) vs. no/bad atmosphere	12	•	•	•	•
	Interactions among residents	Mutual help; friendship; lonely/solitary; respect; discussions; capacity to adapt to new/different residents; conflicts	10	●	•	•	•
	Resident-staff interactions	Trust (bidirectional); appropriate language; respectful; distance; egalitarianism; attachment; listen/understand; adapt to the person; availability	13	●	•	●	•
**Rules and functioning** **(management practices)**	Residents' participation	To be allowed to: do tasks (e.g., cooking); decorate bedroom and common areas; make suggestions (e.g., menu, rules); residents' meetings (committee)	12	●	•	•	•
	Restrictive practices and level of choices	Leave freedom; do not impose; do not tell what to do; rigidity To be allowed: to take drugs/alcohol; to receive visitors; to have a sex life; to make choices; to lock bedroom; to lock bathroom; to access kitchen or refrigerator	13	●	•	•	•
	Rules and regulations	Importance/existence of rules (e.g., schedule, curfew); flexibility of rules; clearly articulated; application of *Quebec Civil Code* only (no other explicit rules)	13	•	•	•	•
**Support to service users**	General help and support	Multiple sources (e.g., peer support, social network, services external to CRF or peripatetic); offer appropriate services; active support; support goals and treatment/recovery plan	13	●	•	•	●
	Spirituality	Respect and encourage spirituality and values	5	●		•	•
**Person**	Emotions	Moral and emotional support; understand/talk about problems	12	●	•	•	•
	Personal growth	Develop potential; support recovery; build a life for oneself	12	•	•	•	•
	Variety of forms (array and intensity)	Do with person; do for person; have the person do on his/her own; develop potential, skills training, motivate, reinforcement, stimulate, provide feedback, encourage, teach, supervise, accompany, etc.	13	•	•	●	•
**Clinical** **and rehabilitation activities**	Domestic activities and activities of daily living (ADL)	Medication, personal hygiene, dressing, healthy lifestyle; diet; mental and/or physical health; budget; purchases; cigarette management; medical appointments; transportation	13 12	• ●	• •	• •	• •
		Meal preparation; laundry; housework; groceries					
	Occupations Group activities (outings) Physical activities Employment	Meaningful occupations; celebrations (e.g., holidays, birthdays), offer daytime activities and workshops in CRF; explore interests Offer group activities outside CRF (e.g., movies) Encourage/offer physical activities Support for finding/maintaining work	13 5 5 11	● ● • •	• • •	• • • •	• • •
	Community integration	Encourage community integration; accompany; explore community resources	9	•	•	•	•
	Social skills/network	Support interactions with family/friends; mediate/manage conflicts between residents; support for sex life; encourage/teach personal expression; offer support to residents' families	10	•	•	●	•
	Provide information	On: rights, health, diseases and symptoms, sexuality, recovery; medication and alternatives	8	●	•	•	•
	Transition and integration	Prepare placement and integration in CRF; support during transition; discuss grief, loss and integration; help with moving; introduce to neighbourhood; welcome residents	12	●	•	•	•
	Independent living	Help with finding/renting an apartment; finding roommate(s); support in supported housing (lack of)	11	●	•	•	●

CRF, Community-based residential facility; U, Service users; F, Family members; M, Managers, administrators and professionals supervising residential settings; S, Staff working in residential facilities and mental health workers. A larger dot indicates a higher occurrence of statements for a stakeholder group (sub-theme/code predominance). A smaller dot indicates a sub-theme/code generated by a stakeholder group. No dot indicates no statement emerged for a stakeholder group for a sub-theme/code.

Results reveal convergence among stakeholders; out of the 50 sub-themes/codes only six (12%) were not mentioned by all four stakeholder groups and most (37/50, 74%) were mentioned in at least 10 of the 13 brainstorming sessions. Some were mentioned predominantly by specific stakeholder. Statements regarding quality of care and management were more frequent among managers. Families brought up their participation as partner and their need for support. Staff advocated for local partnerships, support and ongoing training. For service users, sub-themes regarding social and physical environments, rules and functioning, and clinical/rehabilitation activities were prevalent. No service user statement fell under three out of the five sub-themes of *local partnerships* and under one out of the three sub-themes of *facing stigma*. Service users emphasized sub-themes closer to daily life: interactions, support, making choices, not being imposed upon, and participation in own life and in life inside and outside the setting. Occupations also emerged as a central sub-theme (*occupations, group activities, physical activities, employment, ADL*). Recurring sub-themes among service users also included the involvement of *family members* as well as *individual outcomes* which serves as an indication of what is expected from this complex intervention by stakeholders. Codes related to this sub-theme/code were nevertheless considered irrelevant based on our research questions and were removed in the process of creating the final list of 140 statements. *Conflicting values/incoherence* is another example of a sub-theme that was excluded.

### Statements Relative Importance

The relative importance of the selected 140 statements ranged from 2.71 (1.15) to 4.79 (0.45) (n = 416); most distributions were negatively skewed. Two-thirds of the 140 statements (93/140, 66%) had a perceived importance of 4 or higher (4 = important). Three statements (2.14%) were rated below 3 (3 = more or less important). Only a few statements (15/140, 11%) were significantly rated differently by the two sets of participants. However, only 62/172 (36%) of the service users managed to rate all statements during the 2- to 2.5-h group session. Of these, 25 (40%) were living independently. Consequently, completion was significantly linked to living arrangement (χ2 = 39.68, df = 3, ρ = 0.000).

### Computer-Generated Point Map and Cluster Map

**Figure 1:**
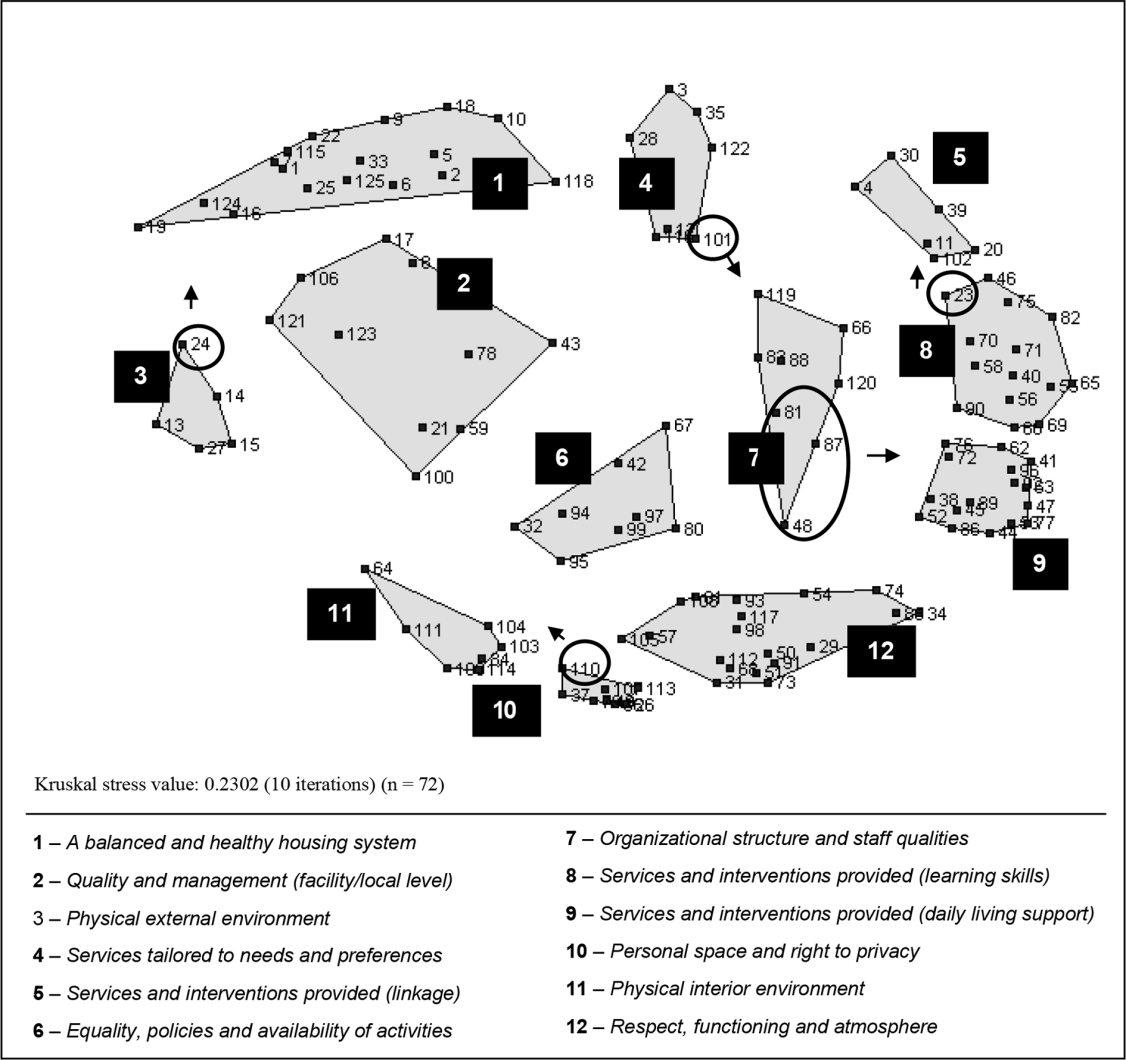
Computer-generated two-dimensional cluster map of the attributes of community-based residential facilities (number of statements = 125).

The 125 printed statements were grouped on average into 10.11 (SD = 4.16) clusters by participants (range = 2-20). The number of statements per cluster varied from 5 to 25 and averaged 11.67 (SD = 6.75). Based on the piles of statements made by participants, the MDS analysis produced an interpretable point map that displayed the 125 statements. Each of the 125 statements are indicated on the map by a dot and number (see dots in **Figure 1**). More similar or related statements are located nearer each other on the map, reflecting a high degree of conceptual similarity as judged by participants. Distance between dots would not change if the map was rotated or if clusters were modified. The stress value for the two-dimensional solution is 0.23 after 10 iterations and indicates a good representation of the participants' sorting (excellent correspondence between the model represented and the similarity matrix on the basis of concept mapping guidelines) (39, 50).

The original computer-generated cluster map configuration is shown in **Figure 1**. The cluster map consists of polygon-shaped boundaries on top of the point map. The research team selected the 12-cluster solution. The 13-cluster solution divided cluster 1 into 2 distinct clusters, but their content overlapped. The 11-cluster solution regrouped clusters 11 and 10 which appear to bring distinctive elements into the conceptualization. **Table 3** presents the 12 clusters along with each cluster average bridging index, random examples of statements, and statement mean importance ratings for each stakeholder subgroup. The statements and the clusters in **Table 3** can be identified on the map by their corresponding number (no). Bridging index range from 0.00 (statements no 52, 45, 62, and 72) to 1.00 (statement no 27). Clusters 2, 4, and 3 comprise relatively diverse statements with an average bridging index above 0.50

**Table 3:** Random statements from the final 12 clusters and importance ratings (mean) by stakeholder groups.

n°	Cluster (C) label and statements (average bridging index, SD)	n ^b^	Mean importance (*SD*) Stakeholder groups ^a^			
			S1		S2	
			U^c^	F	M	S
	**C1 – A balanced and healthy housing system (0.43, 0.13)**	25				
2	*Have training programs for operators and staff working in community-based residential settings*		–	4.91 (0.30)	4.59 (0.57)	4.69 (.439)
9	*Have rapid access to housing (avoid long waiting lists)*		4.33 (0.93)	4.73 (0.47)	4.61 (0.52)	4.73 (0.46)
33	*Have flexible and non-restrictive admission criteria for residential facilities (include consumers who use alcohol and drugs)*		3.84 (1.07)	3.73 (1.22)	3.75 (0.94)	3.92 (1.04)
10	*Ensure good fit prior to integration in setting (make sure setting's attributes fit the person's characteristics)*		4.58 (0.59)	4.73 (0.47)	4.79 (0.41)	4.70 (0.53)
	**C2 – Quality and management (facility/local level) (0.53, 0.11)**	12				
78	*Maintain staff stability (limit turnover)*		4.11 (1.05)	4.36 (0.51)	4.31 (0.74)	4.86 (0.51)
106	*Adapt the physical environment of setting (accessibility and safety features)*		4.10 (1.00)	4.36 (0.67)	4.53 (0.57)	4.57 (0.65)
43	*Give consumers a handbook outlining facility policies, functioning and available services inside and outside the setting*		3.93 (1.08)	4.09 (0.70)	4.35 (0.66)	4.07 (1.03)
	**C4 – Services tailored to needs and preferences (0.52, 0.09)**	6				
12	*Modify support and services according to each service user's needs and condition (consumer does not have to move in event of gain or loss of functional autonomy)*		4.70 (0.49)	4.64 (0.51)	4.23 (0.75)	4.37 (0.66)
122	*Always ask the person where she or he wants to live first*		4.41 (0.80)	4.45 (0.52)	4.66 (0.55)	4.54 (0.54)
	**C3 – Physical external environment (0.83, 0.11)**	5				
14	*Have easy access to resources and services (e.g., grocery store, bank within walking distance)*		4.53 (0.71)	4.27 (0.65)	4.40 (0.61)	4.44 (0.63)
27	*Live in a normalizing neighborhood (i.e., access to leisure and services regardless of severity of illness)*		3.94 (1.02)	4.18 (0.60)	4.50 (0.57)	4.46 (0.64)
136	*Live in a safe and secure neighborhood (e.g., low criminality)*		4.23 (1.00)	4.18 (1.08)	3.38 (0.91)	3.61 (1.14)
	**C11 – Internal physical environment (0.30, 0.14)**	9				
110	*Live in an attractive, comfortable, clean environment*		4.68 (0.51)	4.36 (0.51)	4.61 (0.52)	4.56 (0.61)
111	*Have common areas in setting (e.g., kitchen, living room)*		4.65 (0.58)	4.09 (0.54)	4.25 (0.74)	4.45 (0.57)
	**C10 – Personal space and right to privacy (0.12, 0.08)**	7				
113	*Have access to a telephone in a private space*		4.51 (1.00)	4.36 (0.67)	4.56 (0.61)	4.54 (0.64)
37	*Be allowed to have sexual objects in privacy of own bedroom (e.g* ***.*, ** pornographic magazines)		4.55 (0.76)	3.70 (0.95)	4.15 (0.78)	4.11 (0.87)
79	*Be allowed to refuse to participate in activities organized by setting*		4.31 (0.83)	4.00 (0.78)	4.47 (0.62)	4.33 (0.70)
	**C12 – Respect, functioning and atmosphere (0.09, 0.05)**	21				
112	*Live in a warm, humane setting*		4.06 (0.94)	4.55 (0.74)	4.74 (0.47)	4.56 (0.60)
51	*Create a setting where each person feels respected (e.g., civility)*		3.72 (1.02)	4.27 (0.91)	4.91 (0.28)	4.91 (0.22)
	**C 6 – Equality, policies and availability of activities (0.24, 0.09)**	8				
97	*Have clear, appropriate sanctions*		4.27 (0.93)	4.10 (0.88)	3.86 (0.96)	4.23 (0.72)
99	*Promote equality between staff and service users*		4.11 (0.96)	3.73 (1.27)	4.16 (0.80)	4.32 (0.86)
	**C8 – Services and interventions provided (skills) (0.18, 0.08)**	13				
56	*Assist/teach the person how to use public transit*		3.99 (1.54)	4.09 (1.14)	4.38 (0.61)	4.39 (0.67)
70	*Provide information on recovery and support*		4.28 (1.02)	4.10 (0.74)	4.44 (0.65)	4.48 (0.60)
71	*Provide information on citizenship and rights*		3.60 (1.44)	3.82 (0.87)	4.50 (0.57)	4.42 (0.69)
	**C5 – Services and interventions provided (linkage) (0.38, 0.05)**	8				
20	*Work together to support person's treatment and recovery plan (staff working in facility, community mental health workers, family, service users)*		4.50 (0.645)	4.82 (0.41)	4.74 (0.52)	4.77 (0.45)
4	*Provide support to service users' families*		3.98 (1.11)	4.73 (0.65)	4.21 (0.74)	4.32 (0.71)
	**C9 - Services and intervention provided (daily support) (0.05, 0.04)**	20				
52	*Provide reminders and help with hygiene*		4.63 (0.52)	4.55 (0.52)	4.36 (0.71)	4.66 (0.55)
44	*Supervise daily domestic activities (e.g., laundry, dishes)*		3.87 (1.14)	4.09 (0.54)	3.92 (0.62)	4.14 (0.68)
63	*Use an approach that focuses on consumer strengths and capacities*		4.42 (0.83)	4.60 (0.52)	4.78 (0.53)	4.81 (0.41)
	**C7 – Organizational structure and staff qualities (0.24, 0.10)**	6				
119	*Encourage interventions by peer-support workers*		3.77 (1.19)	4.18 (0.75)	4.18 (0.74)	4.03 (0.83)
88	*Have staff who believe in each individual recovery process and hopes*		4.21 (1.06)	4.73 (0.47)	4.76 (0.53)	4.69 (.439)
101	*Have staff with knowledge of issues related to mental health*		4.32 (0.89)	4.82 (0.41)	4.53 (0.55)	4.73 (0.46)

a U, Service users; F, Family members; M, Professionals, managers and administrators; S, Staff working in residential facilities and mental health workers; S1 = C+ F and S2 = M + S

b 140 statements (15 statements added in step 5 include statement n° 136)

c n = 62 services users (completed the task)

A review of subgroup comparisons suggests that there were more similarities than differences across cluster maps created for each of the four stakeholder groups. Numerous groupings of statements were consistent across participants, although individuals contributed different labels. For instance, many operators and staff working in residential facilities classified statements in terms of who was responsible for a given matter, for example, the housing agency (cluster 1), staff working in residential facilities (cluster 9), or community mental health teams or peripatetic staff (cluster 8). In other words, stakeholders' position within the system and their lived experiences had more of an influence on how clusters were labeled than on how statements were grouped.

### Cluster Map as Modified by Stakeholders (Step 5)

**Figure 2:**
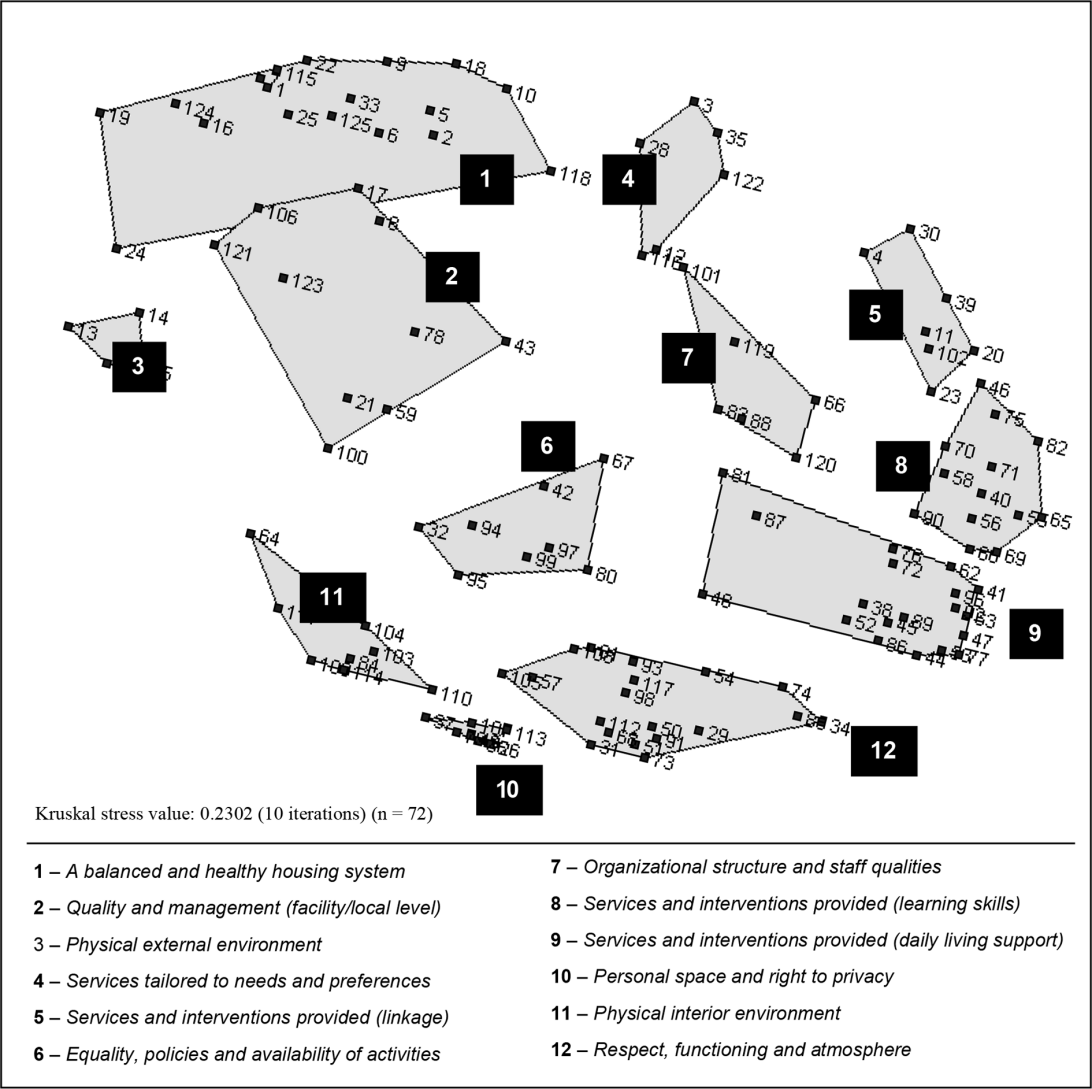
Final two-dimensional cluster map as modified by stakeholders (number of statements = 125) (step 5).

The 12-cluster solution was kept by the participants during the final group session (step 5). The presentation of the map cluster-by-cluster allowed the participants to label each cluster (with a different language than found in the literature). They identified six statements that should be moved to a neighborhood cluster (see circles in **Figure 1**). The final cluster map is presented in **Figure 2**. The participants agreed on the location on the map of each of the 15 additional statements (125 + 15 = 140). For instance, statement no 136 was added to cluster 3 (see **Table 3**), the statement *“Availability of a transparent and simplified procedure to access community-based residential settings and housing”* was added to cluster 1 and “*Access to self-help groups of support for service users*” was added to cluster 5.

Cluster 1 A *balanced and healthy housing system* (18 + 7 additional attributes/statements) focus is on generating a well-coordinated, supportive and efficient system delivering an array of housing options for a variety of service user needs, abilities and preferences. It comprises attributes related to the availability of a range of housing options in terms of services provided, level of supports, lengths of stay, locations, and types of accommodations. Other attributes are related to the availability of sufficient local investments, the availability of trainings for staff and the continuous development of their expertise. Good management leadership, ongoing monitoring and the availability of support for property owner, residential facility managers and staff including during evenings and weekends are also included in this cluster.

Cluster 2 *Quality and management (facility level)* (10 + 2 attributes) is a relatively diverse group of statements with bridging values ranging from 0.42 to 0.74 (M = 0.53) (in other words, participants did not pile them together in a consistent way). It targets quality of care and management practices at the facility or local level. It includes attributes such as staff and mental health workers stability, the availability of a welcoming booklet explaining the basics of the housing functioning and of the neighborhood, the utilization of an anonymous quality of care indicators for family members and service users (survey), and the availability of specific services (e.g., activities in residential facilities, adapting the physical environment to reduce fall risk (e.g., for elderly), menus reviewed by nutritionists).

Cluster 3 *Physical external environment* (4 + 1 attributes) is the smallest cluster. It regroups statements related to the quality of the external physical environment and suggests the importance of aspects of neighborhood such as access to community resources and local stores (proximity to), greenery, availability of public transportation and neighborhood safety and acceptance of individuals with mental illness. This cluster has the highest average bridging index (range = 0.42 to 0.74, M = 0.83) despite the apparent relationships among statements.

Cluster 4 *Services tailored to needs and preferences* (6 attributes) focus is on fit. It comprises attributes describing the provision of flexible services tailored to needs, abilities, and preferences. It emphasizes the evaluation process and access to flexible levels of support (when needed without having to move) including mental health and physical health services. Finally, it addresses the notion that service users' informed choice of living environment has to be accommodated as much as possible.

Cluster 5 *Services and interventions provided - linkage* (7 + 1 attributes) is a reminder of the variety of the actors involved and of the importance of a real partnerships between them: family members, professionals from outside the setting, treating teams, landlords, community associations, and the service user. Two statements concern the availability of support and communication during service user transitions between places of residence. One statement mentions the provision of support to family members (statement no 4, **Table 3**) and another refers to their continuous integration in the care process.

Cluster 6 *Equality, policies and availability of activities* (8 attributes) focus on functioning. It mostly comprises attributes related to rights, a democratic management style [e.g., *To have a residents' committee running in each setting* (…), *To have clear and appropriate sanctions (only when required)* (…)]. Access to activities inside and outside the setting and equality*/*respect between staff and service users are also attributes included in this cluster.

Cluster 7 *Organizational structure and staff qualities* (6 attributes) is about having competent and available staff/mental health workers *who believe in each individual recovery process and hopes*. Statements also comprise to offer the opportunity for peer support services, to reduce staff turnover, and to ensure that staff and operators have competencies in mental health, crisis management, recovery-based practices, and challenging behaviors.

Cluster 8 *Services and interventions provided (learning skills)* (13 attributes) is composed of attributes describing the provision of support aimed at acquiring practical, problem-solving and social skills, and encouraging autonomy: e.g., self-medication, transportation, and budget management, grocery shopping, occupational balance (including employment), emotions, and self-management.

Cluster 9 *Services and interventions provided (daily living support)* (19 + 1 attributes) also concerns services and intervention provided. It encompassed clinical activities, support and interventions as well as treatment orientations adapted to service user abilities and strengths. When compared to statements in cluster 8, the focus is on activities of daily living and making sure that needs are met for service users with different levels of functioning and illness severity: “*To have the service user's budget managed by staff to ensure effective/good management (have money left at the end of the month)*”, “*To have staff in charge of medication to ensure effective management*”, “*To make sure that the person takes a shower once a week*”, “*To do things with the person instead of doing it*”. Statements in this cluster were piled by participants consistently with bridging values from 0.00 to 0.15 (M = 0.05).

Cluster 10 *Personal space and right to privacy* (8 statements) comprises attributes related to space and privacy in the living environment such as *“To have the key to own place”* and *“To be allowed to choose the color and decoration to own bedroom”*.

Cluster 11 *Physical interior environment* (8 + 1 attributes) emphasizes interior appearance and decoration, cleanliness, common areas for service users and visitors, dedicated rooms for staff, and access to a computer/Internet and appliances.

Finally, cluster 12 *Respect, functioning and atmosphere* (19 + 2 statements) contains the most statements. It is about respect (respectful language, politeness, consideration for religious differences) and atmosphere (celebrate birthday, have functional rules, have the possibility of socializing with other residents, have the possibility of eating with others) induced by peers and staff.

## Discussion

To our knowledge, this conceptualization effort is the first one to build on the perceptions and values of multiple stakeholders with a focus on comprehensiveness. Conflicting perspectives between actors, mostly between service users and mental health workers, have been reported in the literature in relation to specific components of care such as housing preferences (2, 52, 53) and atmosphere [e.g., (54)]. The results of our analyses show overall concordance in relation to the attributes to be used to describe the array of community-based residential settings (sub-themes/codes generated by the stakeholder groups in the four regions), attributes relative importance (most statements were important) as well as conceptually (via sorting sub-analysis).

The GCM process was rich and produced numerous statements that were reduced to 140 attributes of housing and community-based residential settings for adults with severe mental illness. The participant-driven visual representation pictures housing as an input or independent variable (10). It suggests that housing should be apprehended and systematically measured beyond the types of housing and the intensity of services provided with a set of common attributes. Although a detailed discussion of each attribute and cluster is beyond the scope of this paper, housing research has addressed most of these over the last five decades. Moreover, the literature includes several tools developed to assess one or several of the different groups of attributes represented in our conceptual model. Also, beyond the scope of this paper, a review of existing instruments was included in the second phase of the research program (38).

### Further Analyses of the Generated Conceptual Model (Step 6)

It became apparent that the location of attributes on the maps could be interpreted in relation to proximity to the individual living in a housing setting (**Figure 2**). Statements in the upper part of the map reflect a broader system perspective. This part of the map regroups attributes related to housing and community-based residential settings at the agency (clusters 1 and 4) or at the facility/local level (clusters 2 and 5). Statements in the lower part of the map are concerned with the service user proximal environment either physical (clusters 10 and 11) or social (cluster 12) as well as with the services and interventions received daily in the milieu and adapted to one's needs, abilities, and strengths (cluster 9). Statements in the middle part of the map are reflective of service user interactions with several actors (mainly staff or property managers: clusters 6 and 7) and with their neighborhood (e.g., cluster 3 in relation to the environment and cluster 8 in relation to services). Indeed, several of the services and interventions comprised in cluster 8 require the person to interact outside of the setting therefore shifting away activities from the setting. Also apparent is the fact that services and interventions are grouped on the right side of the map. On the left side of the map we find attributes generally related to the physical environment. In the middle part of the map (from top to bottom), we find attributes related to the social and organizational environment (management practices and orientations at the agency/region level (clusters 1 and 4) or in a housing setting (cluster 6) and the atmosphere induced by the relationships between peers, staff, or property managers (cluster 12).

Thus, the two-dimensional map can be apprehended using its two axes. The horizontal axis becomes the focus used to characterize the human environment: (1) physical environment; (2) social and organizational environment including relationships, and (3) interventions and services. The vertical axis is the geographical dimension: (1) the immediate setting (micro level); (2) the external setting, including the neighborhood, peripatetic mental health workers, family members, and the community (mezzo level); and (3) the system or housing agency (macro level). This dimension illustrates the interface between several sectors and actors (e.g., community, family, non-profit, and public sectors) as well as the different levels of analysis characterizing this complex health intervention. These levels show apparent congruence with the conceptual framework proposed by Tansella and Thornicroft for mental health services (32) and with the three conceptual models presented in the introduction (26, 27, 29, 31). Our conceptualization represents three out of the four levels comprised in these conceptual models. The person level (service user individual characteristics) was not considered as an attribute of the setting in this study (step 2) but it is included in the conceptual models of Hall, Nelson & Fowler (1987) and of Moos which illustrates the relationship between program and personal factors (27). Our conceptual model although using different labels and groups of attributes (clusters) also shows similarities with the other dimension (horizontal axis). It adds up all of the domains included in the three conceptual models. Interestingly, as two of these conceptual models were developed years ago, our conceptualization suggests that despite the evolution of housing models and approaches the attributes and dimensions as well as the levels of analysis to be used to describe the full spectrum of housing and community-based residential settings for adults with severe mental illness are relatively stable. It also incorporates an ecological perspective.

### Key Elements of the Conceptual Model

This conceptualization is multileveled (geographical dimension—vertical axis) and multifaceted. It includes numerous components; some are independent (e.g., staff qualities, space arrangement) while others subsume several inter-related features (e.g., pleasant and warm milieu). It illustrates the complexity of housing and community-based residential settings, while at the same time keeping a focus on the micro level where the most attributes (brainstormed statements) are located (see lower parts of the map). These micro level attributes outline the fundamental potential influence of the immediate setting on service user outcomes. This is consistent with the fact that housing or the “home” is central to daily-life experience (29) and the fact that attributes at the micro level are thought to have more influence on individual outcomes (55). Our conceptualization illustrates that the immediate setting clearly affords opportunities for social interactions, care and treatment that go far beyond the formal and tangible interventions and support provided. The high number of statements located in the right side of the map also illustrates the fact that housing can vary widely in relation to programming. To better understand and capture this variation and its impact on service user outcomes is essential.

This conceptualization of community-based residential settings suggests that higher-level attributes deserve attention in an area where most research have focused on limited housing attributes or on a specific geographical level (mostly the micro level). Indeed, more research is needed to identify the most effective practices at the different levels of analysis. For instance, the stakeholders identified specific ways in which the system should support operators and staff working in residential facilities (cluster 1), as well as specific staff qualities and managerial practices likely to influence quality of housing programs (clusters 2 and 7) and outcomes for specific groups of service users.

This conceptualization portrays a multi-person system of interactions as well as interdependence and inter-relations at different levels and between clusters. For instance, looking at the provision of services and interventions (clusters 8, 9, and 5) some services are more likely to be provided by community or mental health workers (mezzo level), while others appear to be more proximal, inside the setting (micro level). Services not provided at one level could easily be compensated for at other levels. Inter-relations between clusters are also evident and can be interpreted in terms of statements/clusters distance on the map. For instance, the overlap between clusters 10 and 11 suggests a close relationship between architectural features and privacy. The overlap between clusters 1 and 2 suggests the influence of the system orientations at a local/facility level.

Finally, this conceptualization uses a unique language. Throughout the group sessions, the research team had realized that most family members, service users and staff or operators working in residential facilities did not expressed their ideas using a language typical of a recovery approach. However, they used different terms and gave concrete examples suggestive of recovery practices. They did not talk in terms of quality, governance, restrictiveness; again, they gave concrete examples. We therefore decided to keep words such a “healthy system” instead of replacing it with “effective” to stay true to the voice of the participants. We used this observation to adapt the content of the tool developed during the second phase of the research program (Phase II) and to be reported in a forthcoming publication.

### Limits and Generalizability of the Conceptualization

The external validity of the results is reinforced by the variety of stakeholders and the wide range of settings involved. Concordance between the themes and sub/themes (the coding frame was based on existing literature) and the final cluster map as well as between the emerging conceptual model (map) and existing models reinforces the external validity of the results.

Both the conceptualization and the GCM process have their limitations. First, the choice made by the research team during content analysis when selecting the 140 generated statements might have influenced the conceptualization (38). Another important limit which reduced the amount of analyses made with ratings data is the fact that ratings for the service user stakeholder group represent the perspective of a subgroup of service users (due to missing data) probably with less severe mental health problems. Despite being incomplete the results suggest difference among the service user subgroups depending on living arrangement. However, once the research team could establish that most statements were important, rating data had very limited impact on the conceptual model presented in this paper as the maps were created based on the participant sorts. Finally, because a statistical package was used to compute the map, the research team did not explore a three-dimensional solution or other algorithm (42, 56).

## Conclusion

The results of the present structured conceptualization illustrate the multifaceted and multilevel nature of community-based residential settings through a visual representation that facilitates comprehension. Concept mapping allowed a rigorous and systematic exploration of the attributes of housing and community-based residential settings ranging from high intensity 24-h congregate settings to independent apartments. The mobilization and involvement of multiple stakeholders allowed covering the entire conceptual domain and identifying components of different levels that might exert an influence on quality of care and outcomes. The results remind us that social and physical environment must be studied together and suggest 12 clusters and 2 dimensions that should be included in the operationalization of housing and community-based residential settings for adults with severe mental illness, including a detailed description of the services and interventions provided and of the governance of the housing system. Thus, the conceptual model provides a structure to guide service evaluation. To understand how housing and residential treatment programs influence the outcomes and behaviors of subgroups of service users, these need to be systematically measured.

## Data Availability Statement

The datasets generated and analyzed for this study can be obtained by contacting the corresponding author (AL).

## Ethics Statement

This study involved human participants and was reviewed and approved by seven ethics review boards affiliated with the following health centres: (1) Institut Universitaire en santé mentale de Montréal; (2) Institut Universitaire en santé mentale Douglas; (3) CSSS de la Vieille-Capitale; (4) Hôpital du Sacré-coeur de Montréal; (5) CSSS de l'Énergie; (6) CSSS du Haut Richelieu-Rouville; (7) CSSS du Nord de Lanaudière. The patients/participants provided their written informed consent to participate in this study.

## Author Contributions

AF conceived and designed the project with supervision from MC and AL. AF and the research assistants collected the data and performed the statistical analysis with supervision from MC, AL, and MK from Concept System Inc. AF drafted the manuscript which was commented and reviewed by all authors.

## Funding

The first author held a doctoral training grant from the Canadian Institute of Health Research (CIHR). The research project was supported by CIHR Dissemination Activities (#KDE228601) and the Institut Universitaire en santé mentale de Montréal (Department of Social Integration). The Institut Universitaire en santé mentale was a study site but had no role in the study design, data collection, data analysis and reporting. The views expressed in this paper are those of the authors.

## Conflict of Interest

MK is the president and principal consultant at Concept System Inc.

The remaining authors declare that the research was conducted in the absence of any commercial or financial relationships that could be construed as a potential conflict of interest.
